# Tuning Electronic Properties of Blue Phosphorene/Graphene-Like GaN van der Waals Heterostructures by Vertical External Electric Field

**DOI:** 10.1186/s11671-019-2999-6

**Published:** 2019-05-28

**Authors:** Jingjing Guo, Zhongpo Zhou, Hengheng Li, Haiying Wang, Chang Liu

**Affiliations:** 1 Henan Key Laboratory of Photovoltaic Materials, and School of Physics and Materials Science, Henan Normal University, Xinxiang, 453007 China; 2 Key Laboratory of Artificial Micro- and Nano-structures of Ministry of Education, and School of Physics and Technology, Wuhan University, Wuhan, 430072 China

**Keywords:** Heterostructure, Blue phosphorene, Graphene-like GaN, External electric field, Electronic properties

## Abstract

The structural and electronic properties of a monolayer and bilayer blue phosphorene/graphene-like GaN van der Waals heterostructures are studied using first-principle calculations. The results show that the monolayer-blue phosphorene/graphene-like GaN heterostructure is an indirect bandgap semiconductor with intrinsic type II band alignment. More importantly, the external electric field tunes the bandgap of monolayer-blue phosphorene/graphene-like GaN and bilayer-blue phosphorene/graphene-like GaN, and the relationship between bandgap and external electric field indicates a Stark effect. The semiconductor-to-metal transition is observed in the presence of a strong electric field.

## Introduction

Two-dimensional (2D) materials such as graphene [1], transition metal dichalcogenides (TMDs) [2], black phosphorene (BP) [3], and graphene-like GaN (g-GaN) [4] have been in the spotlight, owing to their fascinating physical properties and potential applications in devices. As a fast-emerging research area, the way in which the heterostructures are assembled from the isolated atoms remains to be an exciting research filed. It is considered as a novel way to construct devices, which integrates the properties of each isolated component with ideal properties applied in nanoelectronics [5, 6]. Due to atomic layers’ interaction [7], these heterostructures possess outstanding properties comparing with the pure 2D materials, and their properties are preserved without degradation when they are bonded together in the layer-by-layer way. To date, many efforts have been made to obtain van der Waals (vdW) heterostructures. It is worth noting that the blue phosphorene (blue-P)-based vdW heterostructures such as blue-P/TMDs [8–10] and blue-P/graphene [11] have attracted increasing attention due to their excellent electronic and optical characteristics.

Among the above-mentioned 2D semiconductor materials, blue-P monolayer has been prepared by epitaxial growth on Au (111) substrates for the first time in 2016 [7]. Z. Zhang et. al. predicted the epitaxial growth of blue-P monolayers on GaN (001) substrates, and proposed an unconventional “half-layer” growth mechanism. It is also pointed out that blue-P is more stable on the surface of GaN (001) due to the chemical affinity between phosphorus and gallium and the good lattice matching [12]. Blue-P, consisting of a vertically corrugated yet single layer of phosphorus atoms, attracts intense research interest due to its superb properties such as sizable bandgap and high mobility [13, 14]. In addition, g-GaN, as a novel 2D material, can be synthesized experimentally by means of a migration-enhanced encapsulated growth (MEEG) technique [15]. Theoretical simulation has shown that g-GaN is a semiconductor with an indirect bandgap, which can be efficiently manipulated by an external electric field [16]. Like other 2D materials, g-GaN can also be hydrogenated and halogenated conveniently. All these studies have shown that g-GaN is an alternative 2D semiconductor for applications in many important fields in the future. The lattice parameter of g-GaN could match well with blue-P, which indicates that blue-P/g-GaN is an ideal material system for the construction of heterostructures, as well as an excellent inserting layer for tuning of their electronic properties by the interlayer interaction. In this regard, it matters to investigate the electronic and optical properties of the blue-P/g-GaN vdW heterostructures. However, few researches have been investigated to study the properties of blue-P/g-GaN vdW heterostructures [17, 18].

In this work, the electronic structural properties and the variation tendency of the bandgap energy (*E*_*g*_) with the vertical external electric field (*E*_ext_) in the blue-P/g-GaN vdW heterostructures are evaluated and conducted by using the first-principles calculations with vdW-corrected exchange-correlation functional.

## Computational Methods

The band structures and electrical properties of the monolayer and bilayer blue-P/g-GaN vdW heterostructures have been investigated using the Cambridge Serial Total Energy Package (CASTEP) [19], which is based on the density functional theory (DFT) [20, 21] in a plane-wave basis set with the projector augmented wave (PAW) method potential [22, 23]. The generalized gradient approximation (GGA) with the Perdew-Burke-Ernzerhof (PBE) [24] function is adopted to describe the electrons exchange-correlation energy. Since the GGA-PAW approximation usually underestimates the *E*_*g*_ of semiconductors, the hybridization functional HSE06 is carried out to correct them. The effect of vdW interaction [25] is described by the Grimme’s DFT-D2 method. Here, a 500 eV cut-off energy for the plane wave basis was set to ensure the convergence of total energy. A vacuum thickness of 20 Å along the *Z* direction of the blue-P/g-GaN heterostructures is added to eliminate the interaction with the spurious replica images. The atomic positions are optimized until the convergence tolerance of the force on each atom is smaller than 0.001 eV/Å. The first Brillouin-zone integration is used by a fine grid of 7 × 7 × 1 for the structure optimization and 21 × 21 × 1 for electronic state calculation.

## Results and Discussion

**Fig. 1:**
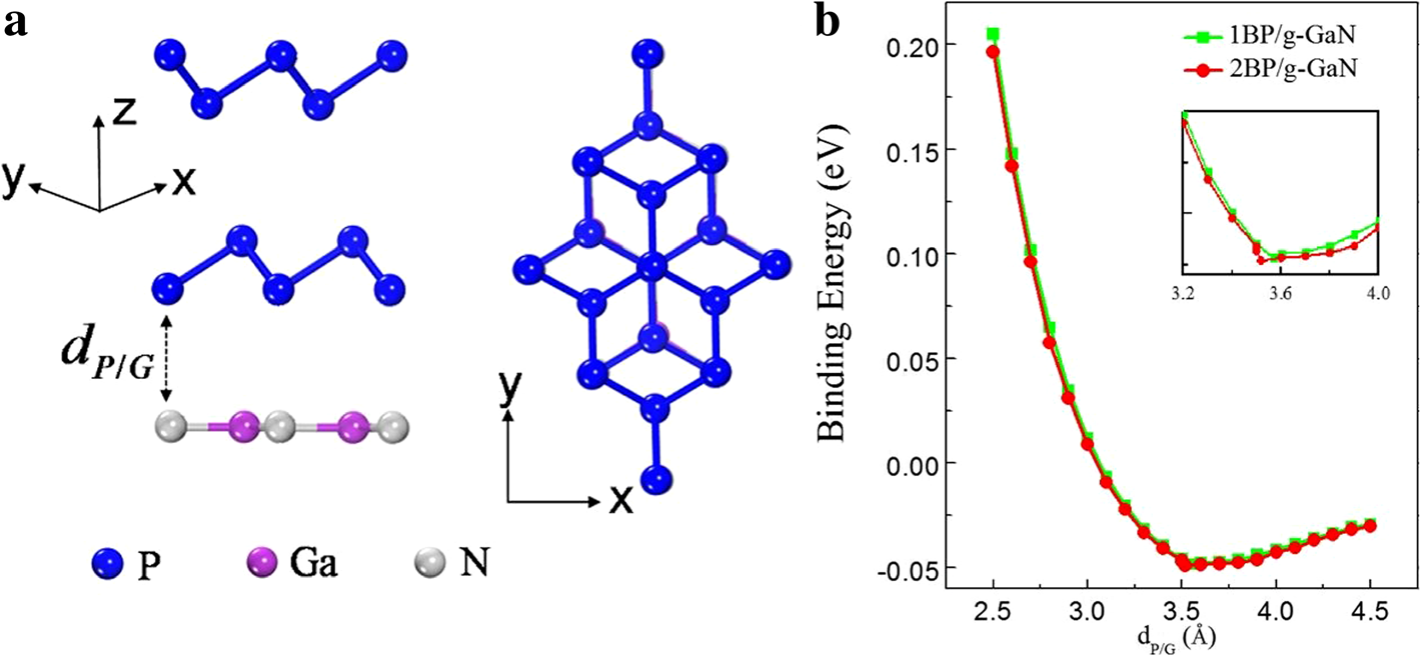
**a** Side and top view of bilayer blue-P on g-GaN. **b** Binding energy as a function of the distance *d*_blue-P/g-GaN_ for the monolayer and bilayer system. The inset shows the zoom close the minimum of the binding energy

Several structures shown in our previous work have been studied as a benchmark to obtain the most stable structure of the bilayer heterostructures [18]. The optimized lattice constants are 3.25 Å and 3.20 Å for bilayer-blue-P and g-GaN, respectively, whose values are in agreement with the reported studies [9, 26]. The lattice mismatch is about of 2% only [18]. In order to obtain the minimum energy configuration and evaluate the thermal stability of the structures, the blue-P layer is moved relating to the g-GaN layer and the lowest energy configuration is found by finite amounts δ_*x*/*y*_. The evolution of the total energy difference as a function of δ_*x*_ and δ_*y*_ is shown in our previous studies [18]. Figure 1a shows the atomic structures of side and top view of bilayer-blue-P on g-GaN. The optimum stacking mode of blue-P bilayers is consistent with the previous paper [27]. Figure 1b demonstrates the relation between the binding energy (*E*_*b*_) at the interface and the interlayer distance of blue-P and g-GaN (*d*_blue-P/g-GaN_). Its definition has been described in detail in our previous studies [18]. The *E*_*b*_ is about 49 meV for the single-layer blue-P with an equilibrium distance of 3.57 Å. For the bilayer, the binding energy is almost the same as that of the single layer, whereas the equilibrium distance is 3.52 Å. Those binding energies have the same magnitude order as other vdW crystals, such as BP/graphene [*E*_*b*_ = 60 meV] [11], blue-P/graphene [*E*_*b*_ = 70 meV] [6], and bilayer blue-P [*E*_*b*_ = 25 meV] [27].

**Fig. 2:**
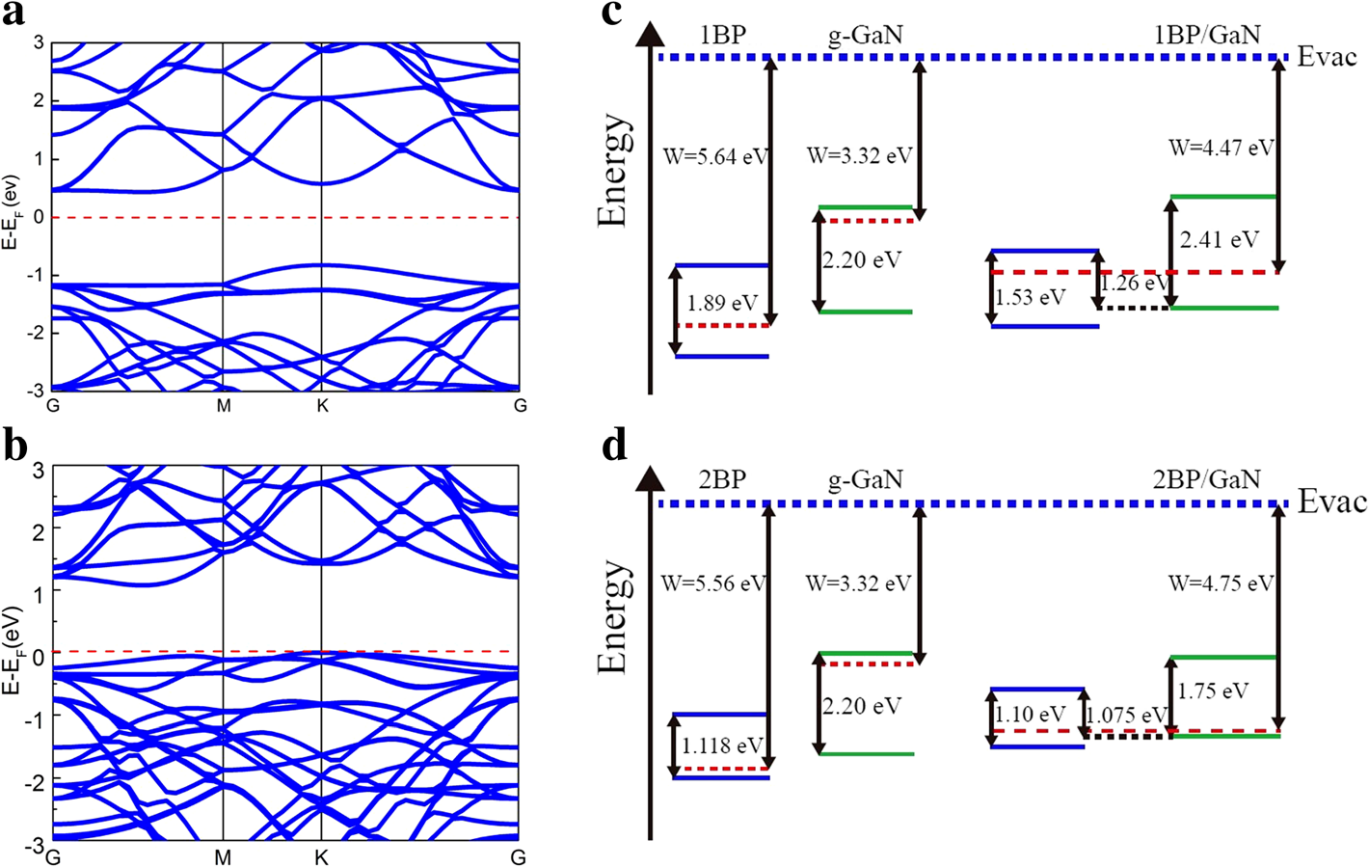
Band structures of **a** monolayer-blue-P/g-GaN heterostructure, and **b** bilayer-blue-P/g-GaN heterostructure, respectively; band alignments and work functions related to **c** monolayer-blue-P/g-GaN heterostructure and **d** bilayer-blue-P/g-GaN heterostructure

Figure 2a-b displays the band structures of monolayer- blue-P/g-GaN heterostructure and bilayer- blue-P/g-GaN heterostructure, with *E*_*g*_ of 1.26 eV and 1.075 eV calculated by using GGA, respectively. For the HSE06 method, the *E*_*g*_ is 2.2 eV and 1.91 eV, respectively. For both heterostructures, the minimal-energy states in the conduction band are near M point and the maximal-energy states in the valence band are at K point, the two points are not at the same crystal momentum in the Brillouin zone. Thus, the bandgap is an indirect band gap for both semiconductor heterostructures. The *E*_*g*_ of monolayer-blue-P/g-GaN heterostructure decreases 0.63 eV compared with the monolayer-blue-P (1.89 eV), while the *E*_*g*_ of bilayer-blue-P (1.118 eV) shrinks 0.043 eV in contrast to bilayer-blue-P/g-GaN heterostructure. The band bending can be achieved from the difference between the Fermi levels of the blue-P with the g-GaN system and the free-standing blue-P [28]: Δ*E*_*F*_ = *W* − *W*_*P*_, where *W* is the work function of the composed system (blue-P/g-GaN), and *W*_*P*_ is the work function of the pristine blue-P. The Δ*E*_*F*_ of − 1.17 eV and − 0.81 eV for the monolayer-blue-P/g-GaN heterojunctions and the bilayer-blue-P/g-GaN heterojunctions are obtained respectively, as shown in Fig. 2c, d. As one can see, the type of the energy band alignment is the staggered gap (type II) at the interfaces for all the monolayer-blue-P/g-GaN heterostructures and the bilayer-blue-P/g-GaN heterostructures.

**Fig. 3:**
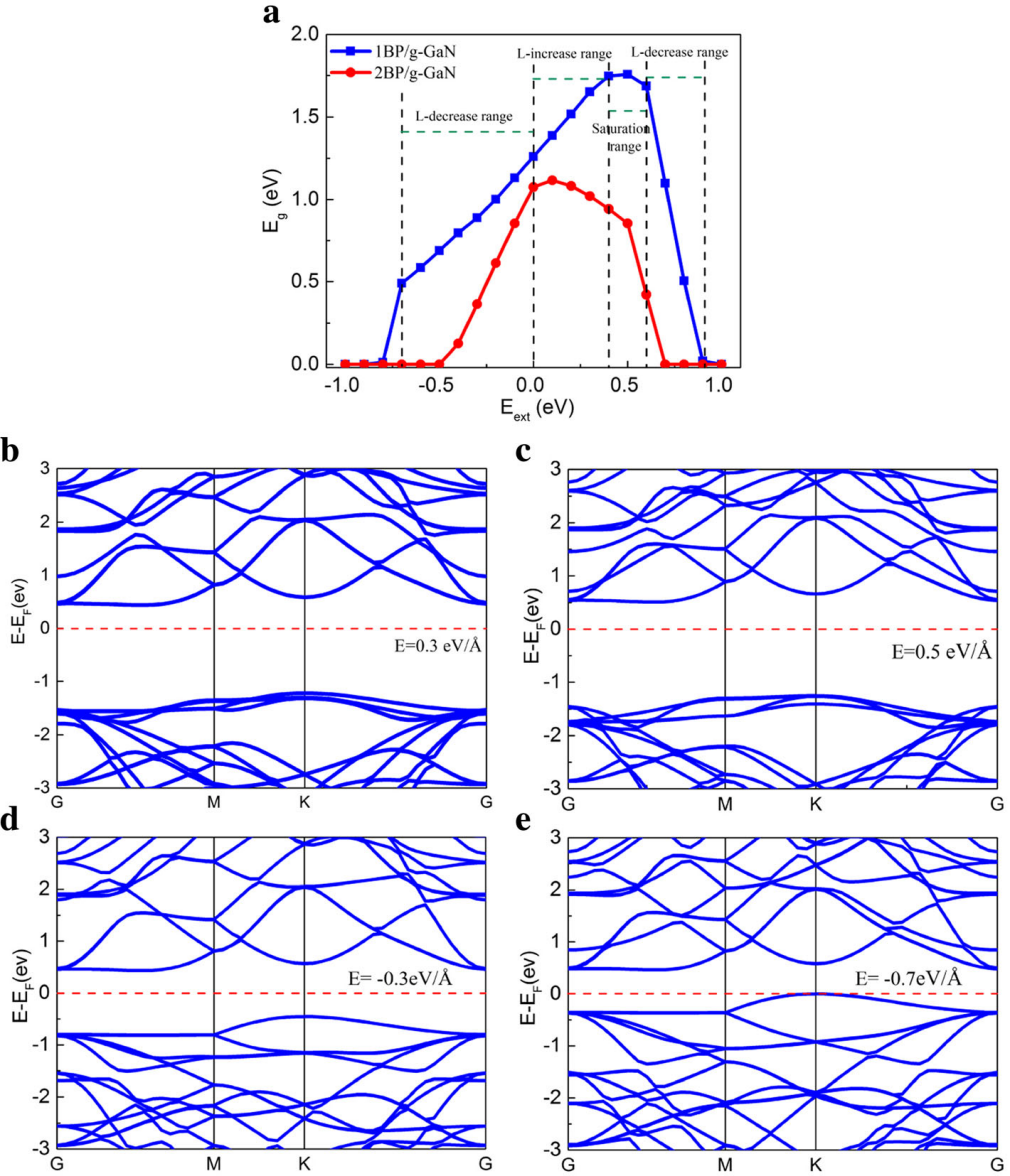
**a** *E*_g_ vs *E*_ext_ of monolayer-blue-P/g-GaN and bilayer-blue-P/g-GaN heterostructures. **b**–**e** The band structures of the monolayer-blue-P/g-GaN heterostructure with *E*_ext_ of 0.3 eV/Å, 0.5 eV/Å, − 0.3 eV/Å, and 0.7 eV/Å. The *E*_*F*_ is set to 0, and indicated by the red dashed line

The heterostructure is often subjected to an external electric field to tune its electronic properties while applied to nanoelectronic devices. In order to study the influence of the *E*_ext_ on the electronic structure, the band structures are calculated with different *E*_ext_ for the blue-P/g-GaN heterostructures. As reported in previous work, the geometrical structure of the heterostructure can be neglected, but the band structure changes greatly under different *E*_ext_ [29]. Figure 3a shows the evolution of the *E*_*g*_ as a function of the *E*_ext_ from − 1.0 eV/Å to 1.0 eV/Å. The direction of *E*_ext_ from top (g-GaN layer) to bottom (blue-P layer) is taken as the forward direction. It is clearly shown that monolayer-blue-P/g-GaN and bilayer-blue-P/g-GaN heterostructures exhibit a bandgap modulation with the *E*_ext_. For monolayer-blue-P/g-GaN, in the case of the forward *E*_ext_, the *E*_*g*_ increases linearly with the increasing *E*_ext_ ≤ 0.4 eV/Å (L-increase range). The monolayer-blue-P/g-GaN obtains its maximum *E*_*g*_ when *E*_ext_ = 0.5 eV/Å and shows little change when *E*_ext_ is in the range 0.4 < *E*_ext_ < 0.6 eV/Å (saturation range), which enhances the band offsets so as to promote the separation of electron-hole pairs. The initial enlargement in *E*_*g*_ is attributed to the counterbalance of *E*_ext_ to some extent by the built-in electric field (*E*_int_). The *E*_*g*_ comes to a linear decrease range with increasing *E*_ext_ > 0.6 eV/Å (L-decrease range). Thus, the heterostructure shows a metal behavior when it is subjected to a stronger electric field. This is originated from the dielectric breakdown as well as charge tunneling. In contrast, the *E*_*g*_ declines linearly with increasing *E*_ext_ (L-decrease range) under a reverse *E*_ext_, caused by the conduction band minimum (CBM) band edge shifting toward to the valence band maximum (VBM). However, when *E*_ext_ = − 0.7 eV/Å, the bandgap begins to decrease sharply, which may be due to the breakdown. When *E*_ext_ < − 0.8 eV/Å, the blue-P/g-GaN heterojunction experiences a transition from semiconductor to metal (metal range). These results reveal that both *E*_*g*_ and semiconductor to metal transition of the blue-P/g-GaN heterostructure is dependent on electrostatic gating, which could be used in high-performance electronic and optoelectronic devices. In addition, the effect of *E*_ext_ on the *E*_*g*_ between the bilayers of blue-P and g-GaN heterostructure is the same as the single layer but with a smaller electronic field for transition from semiconductor to metal.

To explore the effect of electric field on the band structure, the relation between the energy band structures and the external electric field are calculated. The band structures of the monolayer-blue-P/g-GaN heterostructures with *E*_ext_ of 0.3 eV/Å, 0.5 eV/Å, − 0.3 eV/Å, and 0.7 eV/Å are shown in Fig. 3b–e. In Fig. 3b-c, under the 0.3 eV/Å and 0.5 eV/Å of *E*_ext_, the *E*_*g*_ increases to 1.651 eV and 1.757 eV. This indicates the quasi-Fermi level of the g-GaN monolayer is shifted downward, and the quasi-Fermi level of blue-P monolayer is lifted upward. However, in Fig. 3d-e, for the − 0.3 eV/Å and − 0.7 eV/Å of *E*_ext_, the *E*_*g*_ decrease to 0.888 eV and 0.49 eV. The quasi-Fermi level of g-GaN moves upward, and the quasi-Fermi level of blue-P moves downward. The results show that the bandgap varies linearly with the applied vertical *E*_ext_, indicating a giant Stark effect [30]. Upon applying a vertical *E*_ext_, the subband states of the valence and conduction valence would undergo a mixing, leading to a field-induced splitting of the electronic levels. The electrostatic potential difference induced by the external field considerably changed the electronic structures near the Fermi level [31].

**Fig. 4:**
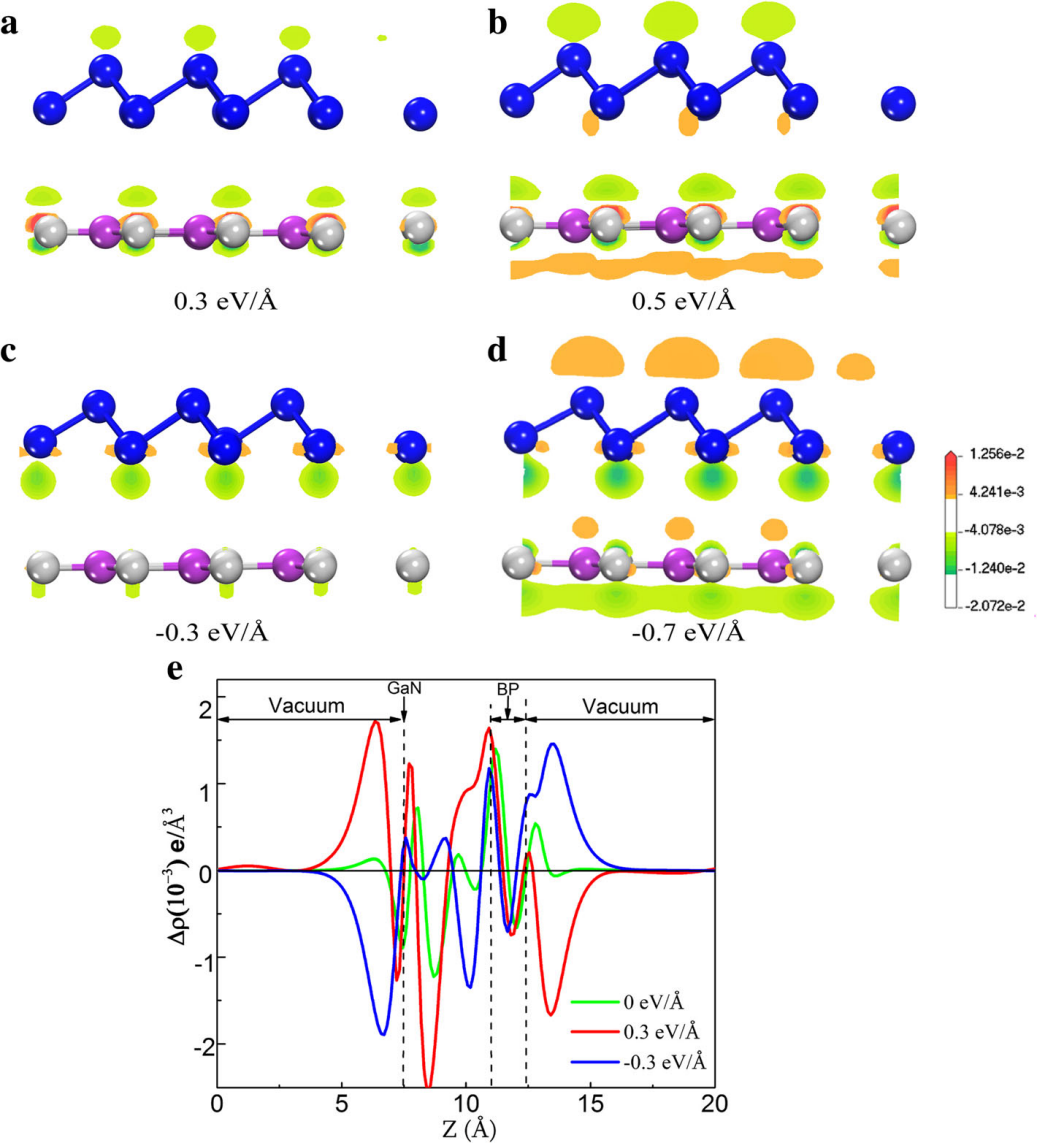
**a**–**d** Isosurface of charge accumulation and depletion of monolayer-blueP/g-GaN heterostructure under *E*_ext_ of 0.3 eV/Å, 0.5 eV/Å, − 0.3 eV/Å, and − 0.7 eV/Å, respectively. Orange and light green isosurfaces represent positive charge accumulation and charge depletion, respectively. **e** Planar-averaged electron density Δρ(*z*) at different electrical field for monolayer-blue-P/g-GaN

 Figure 4a–d shows the isosurface of charge accumulation (with color in orange) and depletion (light green), which exhibits the change of charge density of the blue-P/g-GaN heterojunction with the *E*_ext_ value of 0.3 eV/Å, 0.5 eV/Å, − 0.3 eV/Å, and − 0.7 eV/Å, respectively. Upon applying a forward *E*_ext_, as exhibited in Fig. 4a-b, positive charges (holes) tend to transfer from blue-P layer to g-GaN layer, and negative charges (electrons) transfer from g-GaN to blue-P layer. At the same time concurrently, one can see that the charge-transfer amount is more than 0.3 eV/Å when the electric field is 0.5 eV/Å. Essentially, a positive external electric field orients the charge along the direction of the stress field, restricting the charge to the atomic plane, but leaving the charge in these planes, thereby facilitating the transfer of the charge from blue-P to g-GaN. In contrast, the negative *E*_ext_ induces electrons to accumulate/deplete at the opposite side, as visualized in Fig. 4c-d. Mainly negative external electric fields position the charge back towards the stress field and thus transfer the charge from g-GaN to blue-P. Accordingly, the quasi-Fermi level of g-GaN monolayer and *E*_VBM_ rise, while the quasi-Fermi level of blue-P monolayer and *E*_CBM_ decrease, resulting in a linear reduction on bandgap. Simultaneously, electrons are transferred from blue-P to g-GaN under a reverse *E*_ext_. It is found that the amount of the transferred charge increases with the increase of electric field intensity.

To make it clear that how *E*_ext_ modulates the electronic property, the integrated charge density difference of the monolayer-blue-P/g-GaN heterostructure as a function of the perpendicular distance is calculated, displayed in Fig. 4e. The positive values in Fig. 4e indicate charge accumulation, and the negative values represent charge depletion. For *E*_ext_ = 0, the charge density difference of the heterostructure is obtained by ∆ρ = ρ_heterostructure_−ρ_g-GaN_−ρ_blue-P_. The change of the plane-average charge density difference at interfaces indicates that the electrons were transferred from the g-GaN layer to blue-P layer across the interface, whereas the holes remained in the g-GaN side. The surface averaged differential charge with an electric field is calculated for 0.3 eV/Å and − 0.3 eV/Å. The *E*_ext_ can exert influence on transferring charges in the heterostructure. It can be described as [29]

$$ \Delta  \rho {E}_{\mathrm{ext}}(z)=\int {\rho}_{E_{\mathrm{ext}}}\left(x,y,z\right) dxdy-\int {\rho}_{E_0}\left(x,y,z\right) dxdy $$

where $\int {\rho}_{E_{\mathrm{ext}}}\left(x,y,z\right) dxdy\ \mathrm{and}\int {\rho}_{E_0}\left(x,y,z\right) dxdy$ are the charge density at (*x*, *y*, *z*) point in the supercell of the monolayer-BP/g-GaN heterostructure with and without *E*_ext_, respectively. The direction of charge transfer induced by the negative (blue line) *E*_ext_ is opposite to that of the positive (red line) *E*_ext_. The integrated charge density quantitatively illustrates that the amount of transferred charges increases with the strength of the *E*_ext_. The value of the charges transfers for the blue-P/g-GaN heterostructure with 0.3 eV/Å of *E*_ext_ is larger than that of 0 eV/Å and − 0.3 eV/Å, because the positive external electric field localizes the charges along the direction of the applied field, confining the charges to g-GaN planes.

**Fig. 5:**
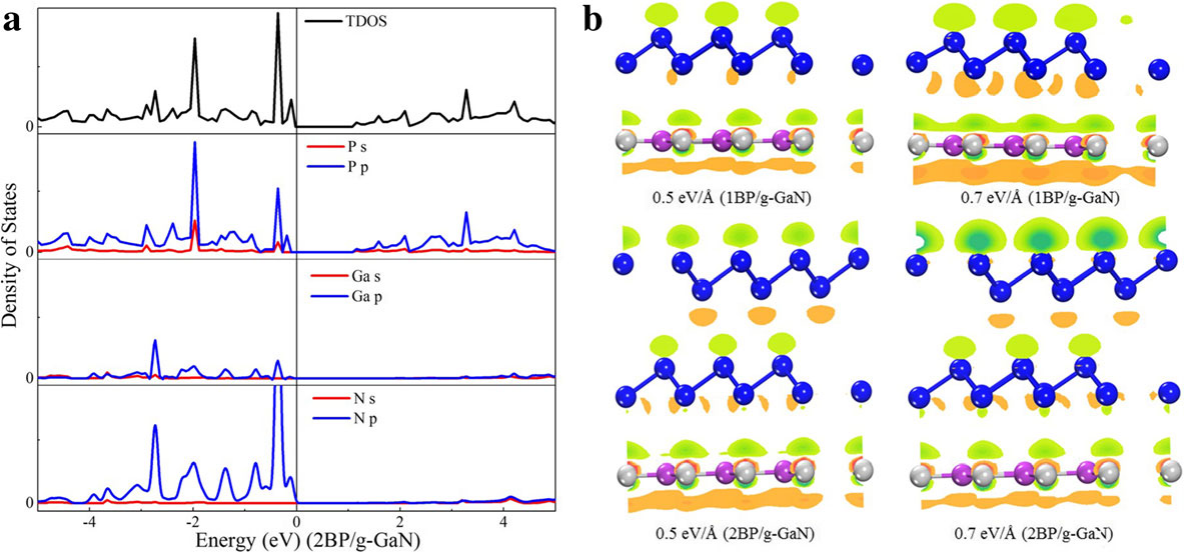
**a** TDOS of bilayer-blue-P/g-GaN heterostructure. PDOS of P, Ga, and N in heterostructure. **b** Isosurface of charge accumulation and depletion of monolayer-blue-P/g-GaN heterostructure under *E*_ext_ of 0.3 eV/Å, 0.5 eV/Å, − 0.3 eV/Å, and − 0.7 eV/Å, respectively

In order to distinguish the contributions of blue-P and g-GaN in the band structure, the projected state density of the heterostructures is calculated and shown in Fig. 5a. It can be seen that the contribution of VBM mainly comes from the g-GaN, and the entrainment contribution is mainly from the blue-P. Figure 5b displays the isosurface of charge accumulation and depletion of the monolayer- blue-P/g-GaN and bilayer-blue-P/g-GaN under 0.5 eV/Å and 0.7 eV/Å external field, respectively. Due to the dielectric breakdown of the bilayer-blue-P/g-GaN at 0.7 eV/Å external field, the current relathed the charge transfer would have saturated under the increasing external field, which is in accordance with that in Fig. 3a.

## Conclusion

In summary, the structural and electronic properties of the monolayer-blue-P/g-GaN and bilayer-blue-P/g-GaN vdW heterostructures are investigated by using first-principle calculations. The results show that the monolayer-blue-P/g- GaN heterostructure is an indirect band gap semiconductor with intrinsic type II band alignment. The band offset and *E*_*g*_ of monolayer-blue-P/g-GaN and bilayer-blue-P/g-GaN can be continuously tuned by *E*_ext_, and the relation between *E*_*g*_ and *E*_ext_ indicates a Stark effect. The *E*_*g*_ becomes zero at − 0.8 and 0.9 eV/Å for monolayer-blue-P/g-GaN, and − 0.5 and 0.7 eV/Å for bilayer-blue-P/g-GaN, indicating a transition from semiconductor to metal.

## Abbreviations

2D: Two-dimensional
Blue-P: Blue phosphorene
BP: Black phosphorene
CASTEP: Cambridge Serial Total Energy Package
CBM: Conduction band minimum
DFT: Density functional theory
GGA: Generalized gradient approximation
G-GaN: Graphene-like GaN
MEEG: Migration-enhanced encapsulated growth
PAW: Projector augmented wave
PBE: Perdew-Burke-Ernzerhof
TMDs: Transition metal dichalcogenides
VBM: Valence band maximum
vdW: van der Waals
